# A Systems Biology Approach on the Regulatory Footprint of Human Endogenous Retroviruses (HERVs)

**DOI:** 10.3390/diseases10040098

**Published:** 2022-11-02

**Authors:** Georgios S. Markopoulos

**Affiliations:** 1 Haematology Laboratory, Unit of Molecular Biology, University Hospital of Ioannina, 45500 Ioannina, Greece; geomarkop@gmail.com; 2 Neurosurgical Institute, Faculty of Medicine, University of Ioannina, 45110 Ioannina, Greece

**Keywords:** endogenous retrovirus, regulatory networks, regulatory elements, systems biology, epigenetics

## Abstract

Human endogenous retroviruses (HERVs) are a family of endogenous retroviruses that comprise the ~8.93% of the human genome sequence, with a high proportion being human specific. The recent expansion of repeated HERV sequences has offered a framework for genetic and epigenetic innovation. In the current report, a systematic approach is implemented to catalogue regulatory elements within HERVs, as a roadmap to potential functions of HERV sequences in gene networks. ENCODE Project has offered a wealth of epigenetic data based on omics technologies. I analyzed the presence of HERV sequences on consensus cis-regulatory elements (cCREs) from ENCODE data. On the one side, HERVs are in 1 out of 9 cCREs (>100.000 cCREs in total), dispersed within the genome and present in cis-regulatory regions of ~81% of human genes, as calculated following gene enrichment analysis. On the other side, promoter-associated HERV cCREs are present adjacent to (in a 200 bp window) the transcription start sites of 256 human genes. Regulatory network production, followed by centrality analysis led to the discovery of 90 core genes containing HERV-associated promoters. Pathway analysis on the core network genes and their immediate neighbors revealed a regulatory footprint that, among others, is associated with inflammation, chemokine signaling and response to viral infection. Collectively, these results support the concept that the expansion of regulatory sequences derived from HERVs is critical for epigenetic innovation that may have wired together genes into novel transcriptional networks with critical roles in cellular physiology and pathology.

## 1. Introduction

A major breakthrough of the Human genome project was that a large proportion of genomic sequence was repeat-associated [1,2]. Based on Repeatmasker algorithm, more than half of the human genome has been calculated to be repeat-associated [3], while other approximations consider that this is an underestimation and over two thirds of the human genome are derived from repetitive elements [4]. Retrotransposons are a major category of repetitive DNA, expanded through evolution mainly through an RNA intermediate, a mechanism called retrotransposition [5]. The mainstream opinion is that expansion of retrotransposons may be deleterious to genomic integrity and lead to disease, that is why the cell has several restraint mechanisms, while in the long term the presence of repetitive DNA can be exploited as a source for genetic novelty [5]. Transposons contain a number of regulatory elements and can regulate the expression of genes within the same regulatory domain [6]. In addition, transposon sequences can affect several physiological processes, such as imprinting, by acting as sites for methylation [7], and recombination, by providing an abundant source of Holliday Junctions sequences [8].

Among repetitive DNA elements, Human Endogenous Retroviruses (HERVs) comprise almost 9% of the human genome [3], having a pan-primate evolutionary distribution [9]. HERVs share the same structural features with exogenous retroviruses and contain Long Terminal Repeats (LTRs), a structural feature for expression and retrotransposition into a novel genomic site. Regulatory sequences derived from HERVs, mostly those present in their LTRs are an abundant source for epigenetic innovation during evolution [10]. 

HERVs contain in their LTRs a rich repertoire of transcription factor binding sites that may be common in different elements or unique [11,12]. Some common transcription factors in many HERV LTRs include among others YY1 [13], Sp1 [14], Myb [15] and NF-κB [16,17]. This polymorphic binding landscape in HERV LTRs may be a reason why different members of HERV family exhibit distinct transcriptional footprints, with a noteworthy upregulation of several members in male and female reproductive system and in early development [18,19,20,21,22,23]. This noteworthy variation of expression is considered that is partly due to the unique features of their LTRs, a source of epigenetic innovation [24]. A well-established paradigm is the role of HERV-H, a primate-specific member of the HERV family that is expressed in naïve-like stem cells [25]. Importantly, sequences derived from HERV-H seem to have a dual role in the epigenetic determination of pluripotency, either by chromatin reorganization during human pluripotent stem cell (hPSC) differentiation, by creating topologically associating domains (TADs) [26], or by their involvement in the evolution of novel enhancers combined with their action as long non-coding RNAs [27].

The results from the ENCODE project have indisputably rejected the idea of junk DNA, since at least one biochemical function has been assigned to the majority of non-coding genomic sequence, including repetitive DNA [28]. On the contrary, this unprecedented wealth of experimental evidence provides a roadmap for discovering novel functions of our genome. Following this conceptual framework, I present a post-genomic view on the regulatory functions of HERV-derived sequences. The results of the current study support a genome-wide function of HERVs in the vicinity of genes as well as a promoter-specific involvement in regulatory networks that may be affecting physiology and pathology.

## 2. Materials and Methods

### 2.1. Sequence Analysis and Annotation

The data retrieval workflow is based in the concepts implemented in previous studies [29,30,31] and included the following steps: A repository on HERV sequences was generated based on repeatmasker tool [3,32] and retrieved using the UCSC Table Browser [33,34,35]. The presence of candidate cis-regulatory elements (cCREs) extracted from data available from the ENCODE (Encyclopedia Of DNA Elements) Project Consortium [36,37] in HERV sequences was determined using the UCSC Table Browser, in the extracted data and further analyzed using bioinformatic tools available in the UCSC Genome Browser and the UCSC Table Browser (http://genome.ucsc.edu/ (accessed on 4 August 2022)) [38,39,40,41]. Table Browser was used to extract the genomic features of HERVs and cCREs, including total number, individual and total size, as well as their relative distributions. The latest GENCODE reference [42] was used to annotate promoter-specific HERV-CRE sequences to their respective gene, in UCSC Table Browser.

### 2.2. Analysis of Genomic Distribution Related to Human Genes

Gene-centered genomic annotation and enrichment analysis was performed using Peak Annotation and Visualization (PAVIS) tool [43], available in National Institute of Environmental Sciences web server (https://manticore.niehs.nih.gov/pavis2/ (accessed on 4 August 2022)). PAVIS offers a gene specific annotation to the following sub-categories: upstream or downstream of genes (up to 1000 bp), 5′ UTRs, exons, introns and 3′ UTRs. 

Total cCREs that contain HERV sequences were annotated in relation to transcription start sites (TSSs) of Human genes using the Genomic Regions Enrichment of Annotations Tool (GREAT) (great.stanford.edu) [44]. The data derived from GREAT were inspected and analyzed further using the UCSC Genome Browser. All the aforementioned servers were last accessed on 4 August 2022.

### 2.3. Network Analysis

Regulatory networks were constructed with the GENEMANIA prediction server tool (www.genemania.org (accessed on 4 August 2022)), using the default settings and provide 20 nearest-neighbor genes in the final network [45]. The initial regulatory network derived from Genemania server was further processed in Cytoscape application, version 3.8.1 [46,47,48]. Centrality analysis to assess the presence of the core genes in the network was performed in Cytoscape, using Centiscape plugin [49,50]. Briefly, the degree of interaction was used as a weighting method to calculate centrality. Central genes with significant interactions were considered the ones that contained a degree of interactions above the threshold of mean interactions.

The participation of the core regulatory network from the aforementioned network in cellular pathways was calculated by enrichment analysis in the Reactome pathway knowledgebase, version 81 (www.reactome.org (accessed on 4 August 2022)) [51]. The Enrichr tool (https://amp.pharm.mssm.edu/Enrichr/ (accessed on 4 August 2022)) [52,53] was implemented to evaluate for enrichment of Gene Ontologies, Cellular Pathways, and Disease. Appyter was used for data visualization [54]. The servers were last accessed on 4 August 2022.

## 3. Results

### 3.1. Overview on HERV-Associated cis-Regulatory Elements

**Figure 1:**
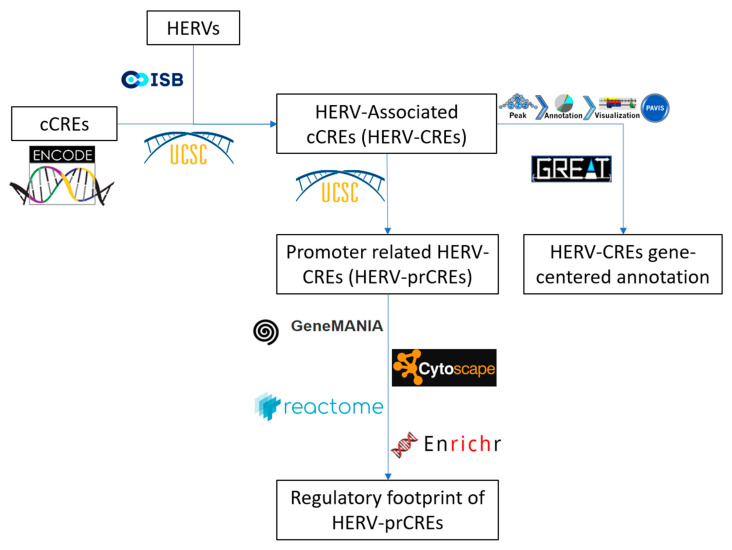
Study overview.

The purpose of the current study was to establish an insight on the regulatory roles of HERV-derived sequences. An overview of the study design is presented in Figure 1. An initial goal was to establish the genome-wide landscape of regulatory regions that are associated with HERVs. First, using the available data from repeatmasker, I filtered for the presence of masked sequences derived from the family of endogenous retroviruses. In total, based on the latest repeatmasker integration of ENCODE data, Human Genome Contains 735.887 HERV-derived sequences that account for the 8.93% of the human genome. Next, I intersected the cCREs extracted from available ENCODE project consortium data (in total 926.535 elements, accounting for 8.14% of the genome), with that of HERVs [55]. As a result, 1 out of 9 cCREs elements (105.875 elements and 0.96% of the genome) contain HERV-derived sequences, later on referred as HERV-CREs. The genomic distribution of HERV-CREs is presented in Table S1. Among the total HERV-CREs, 920 are in promoter-like signatures (PLS), 8.538 are in proximal enhancer-like signatures (pELS), 82.821 are in distal enhancer-like signatures (dELS), while 4.063 are in DNase-H3K4me3 enriched regions and 9.533 are in CTCF-binding sites.

### 3.2. HERV-CREs Are Present in the Vicinity of the Majority of Human Genes

**Figure 2:**
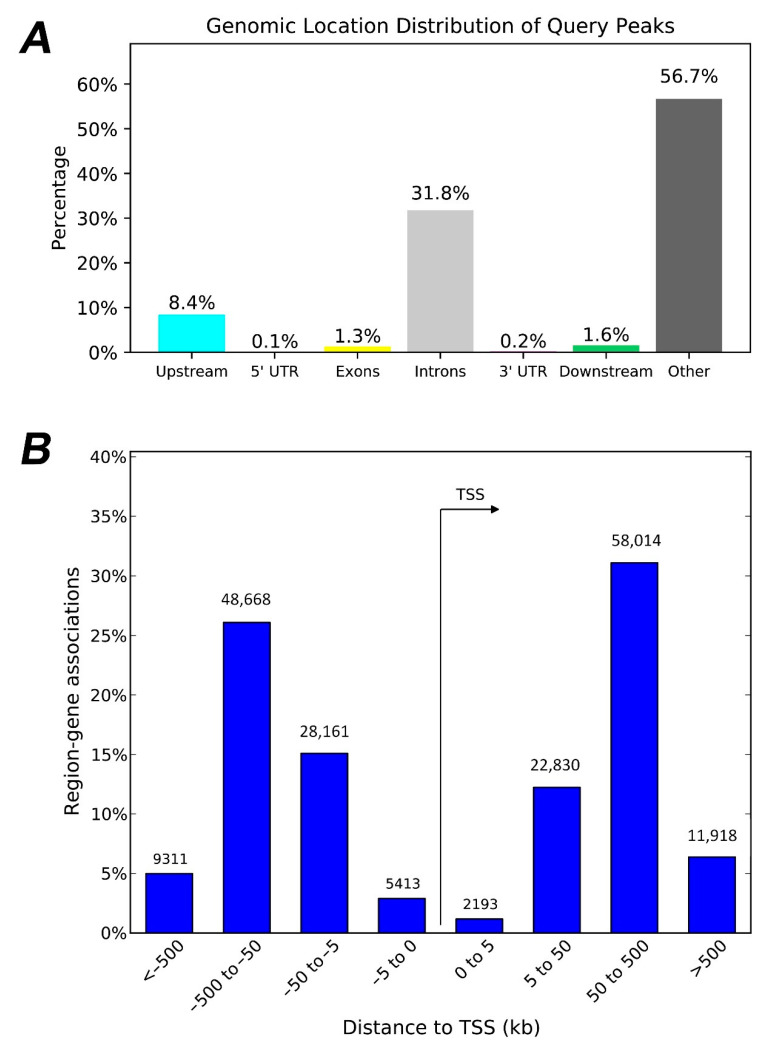
Genomic distribution of HERV-CREs. (**A**) Genomic location distribution in relation to human genes, following PAVIS analysis. The relative abundance of sites upstream, in 5′ UTR, Exons, introns, 3′ UTR, downstream and in intergenic regions is shown in distinct color bars. (**B**) Genomic location distribution in relation to Transcription Start Sites (TSS) of human genes, following GREAT analysis. The number of HERV-CREs in different distances to TSS of mRNA genes is shown. The relative distance to TSS is divided into 0–5, 5–50, 50–500 and >500 kb.

Two types of analyses were performed in HERV-CREs to provide an overview on the genome-wide regulatory landscape of HERV-CREs in relation to human genes. In the first, HERV-CREs were used for enrichment analysis in relation to genes, based on PAVIS tool (Figure 2, panel A). Approximately, 1 out of 11 HERV-CREs reside adjacent to Transcription Start Sites (TSS) of human genes, while the majority is intronic or intergenic, accounting for 31.8% and 56.7% of total HERV-CREs, respectively. In addition, a second analysis was performed to reveal the distribution of HERV-CREs in respect to gene TSS, based on GREAT tool (Figure 2, panel B). The genomic regions of HERV-CREs have been found near the TSS of 15,173 (81%) of all 18,777 genes (Table S2). The majority of HERV-CREs was in a distance of 5–500 kilobases (kb) to the nearest gene TSS. Together, PAVIS and GREAT analysis reveal an interspersed and gene-specific genomic distribution.

### 3.3. The Regulatory Footprint of Promoter-Specific HERV-CREs

Among HERV-CREs, a number of 920 sequences were present in promoter-like signatures (PLS). These elements were further analyzed for their involvement in gene-regulatory networks, since they possess a genomic position in a window of 200 bp near gene TSS, making them the ideal candidates to act as core promoter gene-regulatory elements [56,57]. The UCSC table browser was queried using the latest GENCODE reference to allocate promoter-specific HERV-CREs into their respective genes, using the ENCODE annotation of PLS cCREs, which is regions that fall within a window of 200 bp of an annotated GENCODE TSS. In total, 256 genes were found to contain at least one HERV-CRE in their promoter regions (Table S3). 

**Figure 3:**
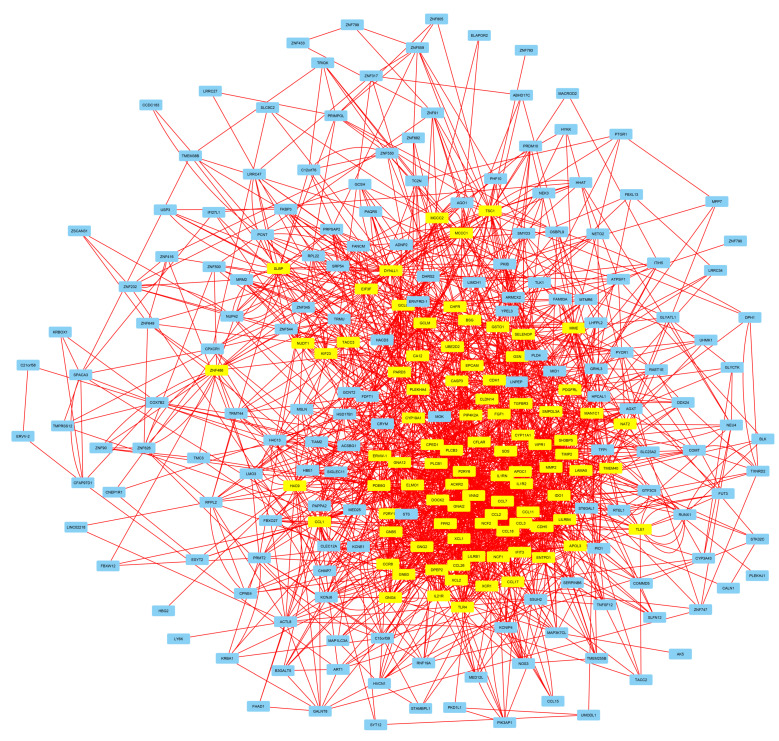
Regulatory network of total genes with HERV-associated regulatory elements in their promoters. Central genes, that contain a significant degree of interactions are denoted in yellow color.

In a latter step, the gene-set containing HERV-PLS CREs, was analyzed in the GENEMania server to generate a regulatory network containing interactions between these genes and a generated dataset of 20 nearest-neighbor interacting genes. The resultant network (containing the genes with at least one interaction and the 20 resultant genes) was transferred into Cytoscape environment for visualization and further analysis with the Centiscape plugin, to find the central/core-network genes, based on the number of interactions. The final network is depicted in Figure 3, indicating the central genes in yellow. 

Additionally, to provide a comprehensive synopsis of the functions on genes in the final network, we added the gene dataset in Reactome server (Table S4). The top-10 enriched cellular pathways, according to enrichment *p*-value, contain: Chemokine receptors bind chemokines; Ca^2+^ pathway; G beta:gamma signaling through PLC beta; Presynaptic function of kainate receptors; ADP signaling through P2Y purinoceptor; Activation of kainate receptors upon glutamate binding; Interleukin-10 signaling; Peptide ligand-binding receptors; G-protein beta:gamma signaling.

**Figure 4:**
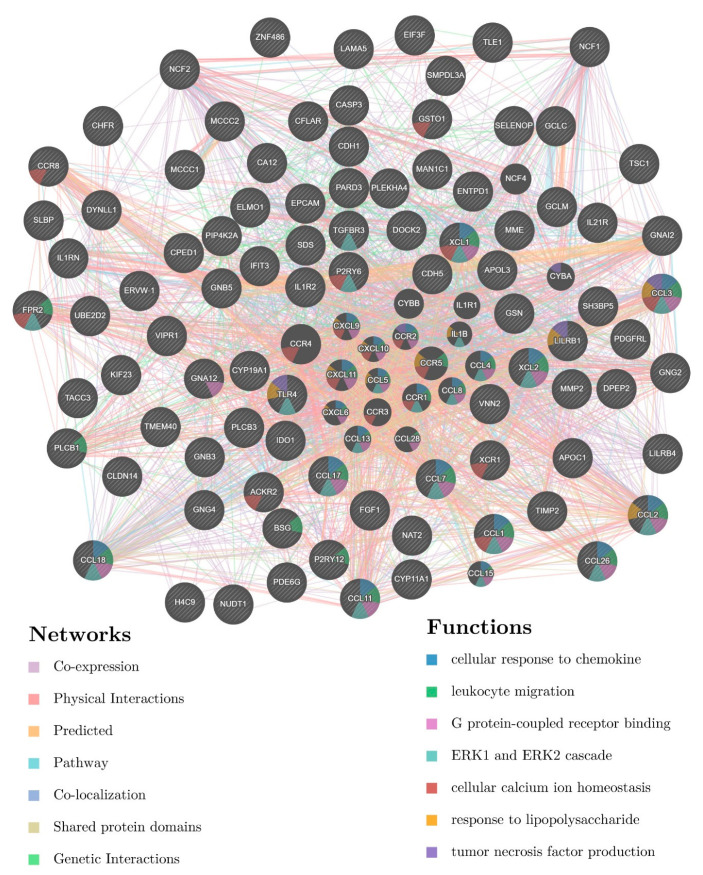
Network analysis of central HERV-PLS CREs genes. The final network includes 90 input genes (indicated with gray stripes) and top 20 related genes, based on GENEMania analysis. The presented, final network, contains 110 genes and 3117 total interactions. Types of interaction are presented as lines with different colors while functions are presented as circles with different colors, representing individual genes/gene products (see the color-coding legend below the network for details).

The calculated network contains 90 central genes, that were further analyzed with GENEMania, to provide the core regulatory network of genes with HERV-PLS CREs (Figure 4). The final, core network contains different types of interactions (Co-expression, Physical Interactions, Predicted, Pathway, Co-localization, Shared protein domains and Genetic Interactions) as well as inferred functions, based on enrichment analysis. Among others, core HERV-associated genes may share these functions: cellular response to chemokine; leukocyte migration; G protein-coupled receptor binding; ERK1 and ERK2 cascade; cellular calcium ion homeostasis; response to lipopolysaccharide; tumor necrosis factor production. In concert, the results from the above analysis provide novel data that suggest that a regulatory network which contains HERV-PLS CREs might exist.

### 3.4. Involvement of Central Promoter-Specific HERV-CREs in Cellular Pathways

**Figure 5:**
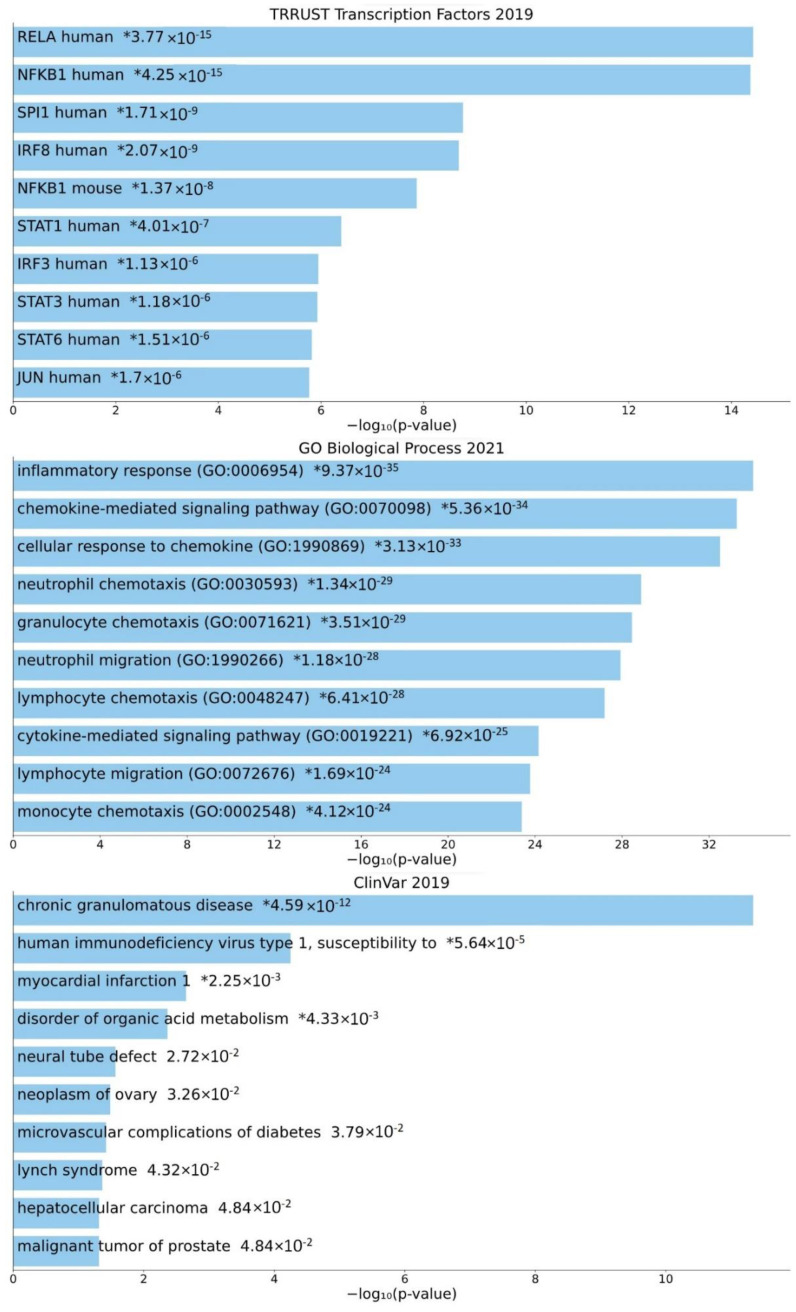
Enriched gene datasets containing HERV-PLS CREs. The dataset of 110 genes, based on GENEMania analysis were used as an input in the Enrichr meta-analysis tool, for enrichment in gene databases. Representative results from KEGG2021 Pathway Human, KEGG2019 Pathway Mouse, Clinvar2019 and MirTarbase2017 are presented. The results were visualized in Appyter tool. * denotes the level of statistical significance.

**Figure 6:**
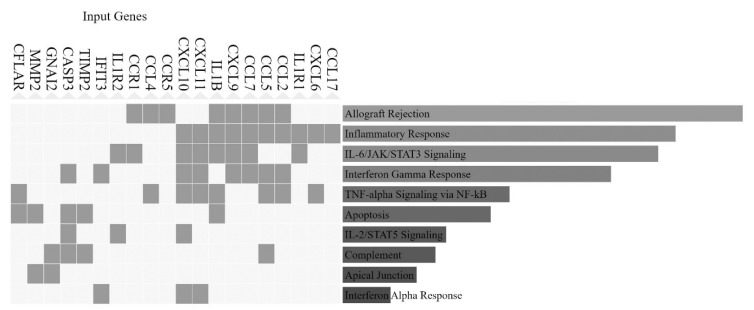
Top enriched HERV-associated genes in cellular pathways, according to MSigDB Hallmark 2020 database. Following Enrichr analysis, the top10 enriched pathways in MSigDB Hallmark 2020 database that contain HERV-associated genes were further analyzed for the existence of common genes. The top20 genes (left panel) were plotted, based on their occurrence in the respective top10 related pathways (right panel).

The associated genes of the central promoter-specific HERV-CREs were further analyzed using Enrichr meta-analysis enrichment tool to assess their association with specific ontologies, pathways and diseases. The results of Enrichr analysis are presented in Figure 5 and Figure 6 and Table S5. Based on enrichment in TTRUST (transcriptional regulatory relationships unraveled by sentence-based text-mining) database, the majority of enriched transcription factors are members of NF-κΒ (including human RELA, and NFKB1) STAT (including STAT1, STAT3 and STAT6) and IRF (including IRF3 and IRF8) families. The top-10 enriched factors also include SPI1 and JUN factors (Figure 5).

Based on the most enriched Ontologies (GO Biological Process 2021), HERV-associated genes are involved in inflammatory response, cytokine and chemokine signaling and the chemotaxis/migration of several types of white blood cells (Figure 5). According to ClinVar 2019 database, central HERV-associated genes are linked to neoplastic disease (such as ovary, hepatocellular, prostate cancer and lynch syndrome), chronic granulomatous disease, susceptibility to HIV-1, myocardial infarction, disorder in organic acid metabolism, neural tube defect and microvascular complications of diabetes (Figure 5).

Importantly, the top enriched cellular pathways for HERV-associated genes according to MSigDB Hallmark 2020 database, contain several common genes. Common genes mostly include members of chemokines, chemokine ligands and their receptors (CCL2, CCL4, CCL5, CCL7, CCL17, CXCL6, CXCL9 CXCL10 CXCL11 CCR1, CCR2 and CCR4), interleukins and their receptors (IL1B, IL1R1 and IL1R2), apoptosis regulators (CASP3 and CFLAR) G-protein GNAI2, matrix metalloproteinase MMP2 and matrix metalloproteinase inhibitor TIMP2. These proteins take part in the enriched pathways: IL-6/JAK/STAT3 Signaling; Allograft Rejection; Inflammatory Response; Interferon Gamma Response; Apoptosis; TNF-alpha Signaling via NF-kB; IL-2/STAT5 Signaling; Complement; Reactive Oxygen Species Pathway (Figure 6).

## 4. Discussion

In this paper a framework on the regulatory roles of Human Endogenous Retroviruses was developed. My approach provides a novel view on gene regulation based on the wealth of data from ENCODE project. The presented conclusions are based on two different approaches. First, a general approach provides an overview of HERV-associated regulatory roles and signifies that 4 out of 5 human genes contain at least one HERV-associated cis-regulatory element. Second, based on promoter-specific HERV elements, I reveal the presence of a distinct regulatory footprint. Genes that form a core regulatory network are involved in significant pathways that impact cellular homeostasis and pathology.

The reference for characterizing a HERV sequence has been the repeatmasker tool [32], a golden-standard approach for transposon characterization. In addition, the cCREs library of regulatory elements from ENCODE [28,37], provided genome-wide experimental data that make possible a post-genomic view of HERV-associated gene regulation with high confidence. An interesting feature of both cCREs and HERVs is their diversity in size and genomic distribution. This diversity is more obvious in cCREs, since there are more cCREs sequences than those of HERVs origin (926.535 elements compared to 735.887, respectively), but cCREs account for a smaller fraction of the genome than HERVs (8.14% of the human genome compared to 8.93%, respectively). Importantly, there is a significant number of cCREs that contain HERV sequences, with the majority belonging to distal enhancer elements. Having that in mind, GREAT analysis revealed that the dispersed nature of HERV sequences allowed for their presence on the regulatory domains of the 80% of human genes (Figure 2). The distribution of HERVs has been verified as genome-wide, as can be seen in elements in the HERV-K and HERV-H groups that some are also polymorphic in human populations [58,59,60]. PAVIS analysis results also suggest an intron- and intergenic region-based distribution (Figure 2). GREAT tool considers a gene regulatory domain definition that takes into account a basal regulatory domain in a minimum distance upstream and downstream of a gene TSS, which is extended up to 1 Mb [44]. This definition comes in agreement with the latest genome-wide experimental and computational data [61,62,63,64,65]. Collectively, the presence of HERVs in cCREs that are based in experimental data, suggest that HERV sequences may impact, in different degree, the epigenetic regulation of 4 out 5 human genes. The fact that HERVs contain an abundance of regulatory elements [66,67,68] and are actively transcribed in humans [69] strengthen the possible impact of the above finding. It should be noted that the regulatory potential of HERVs sequences may also involve viral transcription factors. Interplay between HERVs and several types of viruses, such as tumor viruses [70,71] and HIV-1 [72,73,74], may impact cellular physiology. Since apart from CREs, the presence of trans-regulatory elements may impact the expression of genes and genomic structure [75], future studies are needed to provide a more accurate view on the role of HERVs in the three-dimensional genome landscape. 

Promoter-specific HERVs have a potential to act as core-promoter regulatory elements. Promoter specific CREs were interrogated for the presence of HERV sequences and in a later step they were assigned to their adjacent genes. In total, 256 genes have HERV sequences in their promoters. Based on the concept that repetitive elements from HERVs may act in concert, HERV-related genes have the potential to take part in the same signaling pathways and to form a regulatory network. The inferred network from GENEMania (Figure 4) interconnects HERV-related genes with more than 3.000 interactions, a notion that supports the action of these genes in concert. Several lines of evidence corroborate this notion. First, The action of HERV-H in naïve-like stem cells, where gene are expressed in concert, based on repetitive HERV-H regulatory elements [25]. Second, HERV-K elements can regulate stem cell function and neuronal differentiation [76]. Third, HERV-W have been inferred to regulate inflammatory groups of patients with schizophrenia and bipolar disorder [77]. Among the most significant pathways affected by HERV-related genes, based on Reactome analysis were presynaptic functions as well as GABA B receptor pathway (Table S4). This result is in accordance with pathway analysis where a significant pathway from ClinVar 2019 database is neural tube defect (Figure 5), the functions of HERV-W as well as the fact that brain diseases can be associated with inflammation and HERVs expression [78]. It should be noted that the interactions presented in the regulatory network are based on computational imputations and should be corroborated in future studies. 

Inflammatory response has been surfaced as one among the most significant pathways that are associated with HERV-related genes, based on both Gene Ontology (Figure 5) and MSigDB Hallmark 2020 database (Figure 6). There have been evidence that HERVs are implicated in inflammation [79]. The current study substantiates this concept and reveals a set of genes that have a direct involvement in inflammatory response and immunity (Figure 6), such as chemokines, chemokine ligands and their receptors and interleukins and their receptors [80,81,82,83]. Significantly, the regulatory footprint of HERV-associated promoters contains binding sites for several members of the NF-κB family of transcription factors (Figure 5). TNF-alpha signaling via NF-κB is enriched, while NF-κB family also takes part in Interferon Gamma Response (Figure 6) [84]. NF-κB pathways are among the master regulators of inflammation and immunity [85,86,87,88]. It is known that NF-κB binding sites reside in Alu elements [89] that may act in concert and aid in stochastic gene expression [90]. As regards HERVs, they contain several binding sites for NF-κB factors and their LTRs can be modulated by NF-κB [16,17]. This study suggests the possibility of dispersed sequences from HERVs to act in a similar manner to wire genes into NF-κB-related regulatory networks. NF-κB takes part in regulatory loops and networks with several genes as well as non-coding RNAs and can impact, among others, the interplay between inflammation and cancer [91,92,93,94,95]. This potential supports the notion that such regulatory loops can be also HERV-associated. The core HERV network also include binding sites for transcription factors of the STAT family and enriched pathways of IL-6/JAK/STAT3 Signaling and IL-2/STAT5 Signaling (Figure 6). JAK/STAT pathways are involved in inflammatory response in physiological and pathological conditions [96,97,98]. Based on this study results, I suggest that STAT binding sites may be spread in parallel to that of NF-κB during HERV expansion in the human genome and take part in concerted gene regulation. 

Among the significant pathological conditions in ClinVar, neoplastic disease is present in 4 out of 10 total diseases. HERV genes are associated with ovary, hepatocellular and prostate cancer, as well as lynch syndrome, a hereditary type of nonpolyposis colorectal cancer (Figure 5). The probable involvement of HERVs in cancer is a long-enduring concept [68,99,100]. Specifically, HERV transcripts have been found upregulated in prostate [101], kidney [102] breast [102,103,104,105], hepatocellular [106], ovarian [107] and colorectal cancer [108,109], as well as in soft tissue sarcoma [110] and leukemia [111]. Recent data suggest a connection between HERVs and cancer stemness [112]. In particular, HERV retrotranscriptome has been found to accurately characterize leukemia stem cells [111] and to show a widespread expression that may be tissue- and developmental-stage specific [113]. The aforementioned data agree with the notion that HERV retrotranscriptome can characterize normal and cancer cells, while the question remains whether thay act as the main regulatory elements in such condition or as passive transcribed elements. Consequently, further experimental evidence is needed to provide a causative role for HERVs in carcinogenesis. Previous results from our research team suggest a similar role for VL30s, a mouse analogy to HERVs that can impact a number of cancer-associated pathways in the mouse [30]. Our previous results implicate VL30s in cellular stress and carcinogenesis [114,115,116,117,118], a feature that may be shared with HERVs, based on their similar structure and disseminated genomic distribution. 

Viral infection can lead to transactivation of HERVs, leading to an interplay between exogenous and endogenous viral sequences [70,119]. In this study, HERV-related genes found take part in the susceptibility to HIV-1 pathway (Figure 5). HIV-1 infection activates endogenous retroviral promoters regulating antiviral gene expression [120], a phenomenon with various possible implications for HIV-1 infection outcome, from particle infectivity [121], to release efficiency [122] and activation of immune response [123]. This result is in accordance with the bibliography and supports previous findings. Importantly HIV-1 infection is also associated with NF-κB signaling activation and transposable elements activation [124], which may suggest that HIV-1 and HERVs might be interconnected through a positive-feedback mechanism that involves inflammation induction and NF-κB activation. 

## 5. Conclusions

The intuition of Barbara McClintock led to experimental evidence that introduced the scientific community to the concept of a dynamic genome [125]. Moreover, the components of the dynamic genome have been characterized as controlling elements [126] that may shape organism physiology during evolutionary time, by participating in and forming gene regulatory networks [127]. The current study provides a systemic, post-genomic view on the regulatory footprint of HERV sequences that support the various roles of the dynamic, repetitive part of 9% of our genome.

## Data Availability

The data analyzed are available as links provided in the main text and as supplementary data.

## Supplementary Materials

The following supporting information can be downloaded at: https://www.mdpi.com/article/10.3390/diseases10040098/s1, Table S1: Genomic features of HERV-CREs; Table S2: Genes associated with HERV-CREs; Table S3: Genes with HERV-CREs in their promoter regions. Table S4: Most significant pathways associated with HERV-CREs; Table S5: Total Results following Enrichr Analysis.
