# Supplementary material for: A Systems Biology Approach on the Regulatory Footprint of Human Endogenous Retroviruses (HERVs)
Source: Diseases. 2022 Nov 2;10(4):98. doi: 10.3390/diseases10040098 (PMC9680359; doi:10.3390/diseases10040098)
Supplement: Supplementary file 1 [file diseases-10-00098-s001.zip › Table S4.pdf]

Table S4. Most significant pathways

The following table shows the 25 most relevant pathways sorted by p-value.

Pathway name	Entities				Reactions	
	found	ratio	p-value	FDR*	found	ratio
Chemokine receptors bind chemokines	12 / 57	0.004	4.10e-09	3.60e-06	5 / 19	0.001
Ca2+ pathway	10 / 81	0.005	9.25e-06	0.002	9 / 27	0.002
G beta:gamma signalling through PLC beta	6 / 23	0.002	1.01e-05	0.002	2 / 2	1.44e-04
Presynaptic function of Kainate receptors	6 / 23	0.002	1.01e-05	0.002	1 / 2	1.44e-04
ADP signalling through P2Y purinoceptor 12	6 / 26	0.002	2.00e-05	0.003	4 / 4	2.87e-04
Activation of kainate receptors upon glutamate binding	6 / 34	0.002	8.71e-05	0.011	1 / 6	4.31e-04
Interleukin-10 signaling	9 / 86	0.006	9.06e-05	0.011	3 / 15	0.001
Peptide ligand-binding receptors	14 / 203	0.013	1.04e-04	0.011	7 / 83	0.006
G-protein beta:gamma signalling	6 / 39	0.003	1.82e-04	0.018	12 / 15	0.001
Signal amplification	6 / 41	0.003	2.38e-04	0.021	13 / 19	0.001
RUNX1 regulates transcription of genes involved in BCR signaling	3 / 7	4.62e-04	4.45e-04	0.028	4 / 4	2.87e-04
Post-transcriptional silencing by small RNAs	3 / 7	4.62e-04	4.45e-04	0.028	2 / 3	2.15e-04
G protein gated Potassium channels	5 / 31	0.002	5.10e-04	0.028	3 / 3	2.15e-04
Inhibition of voltage gated Ca2+ channels via Gbeta/gamma subunits	5 / 31	0.002	5.10e-04	0.028	3 / 3	2.15e-04
Activation of G protein gated Potassium channels	5 / 31	0.002	5.10e-04	0.028	3 / 3	2.15e-04
Extra-nuclear estrogen signaling	9 / 111	0.007	5.78e-04	0.028	7 / 39	0.003
Regulation of RUNX1 Expression and Activity	5 / 32	0.002	5.88e-04	0.028	19 / 19	0.001
Adrenaline,noradrenaline inhibits insulin secretion	5 / 32	0.002	5.88e-04	0.028	5 / 6	4.31e-04
Activation of GABAB receptors	6 / 50	0.003	6.70e-04	0.029	8 / 8	5.74e-04
GABA B receptor activation	6 / 50	0.003	6.70e-04	0.029	8 / 9	6.46e-04
G beta:gamma signalling through BTK	4 / 19	0.001	7.06e-04	0.029	3 / 3	2.15e-04
Glucagon-type ligand receptors	5 / 35	0.002	8.75e-04	0.034	2 / 8	5.74e-04
Small interfering RNA (siRNA) biogenesis	3 / 9	5.94e-04	9.18e-04	0.035	3 / 5	3.59e-04

Pathway name	Entities				Reactions	
	found	ratio	p-value	FDR*	found	ratio
**Thrombin signalling through proteinase activated receptors (PARs)**	5 / 36	0.002	9.91e-04	0.035	6 / 16	0.001
**G beta:gamma signalling through CDC42**	4 / 21	0.001	0.001	0.035	5 / 6	4.31e-04

* False Discovery Rate
